# Characterizing orthodontic mini-screws in the hard palate of pigs: An experimental and finite-element study

**DOI:** 10.1016/j.heliyon.2024.e24952

**Published:** 2024-01-22

**Authors:** Cristina Valeri, Angelo Aloisio, Vincenzo Quinzi, Gianmarco di Stefano, Giuseppe Marzo

**Affiliations:** a Department of Life, Health and Environmental Sciences, Postgraduate School of Orthodontics, Università degli Studi dell'Aquila, Via Piazzale Salvatore Tommasi 1, Abruzzo, L'Aquila 67100, Italy; b Department of Civil, Construction-Architectural and Environmental Engineering, Università degli Studi dell'Aquila, L'Aquila, 67100, Italy

**Keywords:** Orthodontic mini-screws, Temporary anchorage devices, Mechanical tests, Pig hard palate, Stiffness, Strength, 3D finite element models

## Abstract

**Objective:**

This paper analyses the mechanical response of mini-implants under pull-out, push-in, and shear forces.

**Materials and methods:**

The authors have devised a specialized testing apparatus, using a universal testing machine, for the mechanical characterisation of orthodontic mini-implants (OMI) installed in pig hard palate. The experimental investigation encompasses seven screw types, each subjected to pull-out, push-in, and shear forces. Each test was conducted three times to assess the inherent uncertainties, with three torque insertions, with a total of 189 tests. The resulting load-displacement curves provide information on the secant stiffness and ultimate capacity of the tested screws in all three loading scenarios.

These findings were used to develop a 3D finite element model to assess the OMI mechanical performance.

**Results:**

Torque is particularly influential in push-in tests for both dependent variables. At the same time, the length appears to be more critical in pull-out tests for the capacity. Diameter's influence is consistent across all tests but is especially significant for the secant stiffness in shear tests.

**Conclusions:**

Pull-out tests displayed linear behaviour until failure, whereas push-in tests showed increasing stiffness with more significant deformation. Higher torque led to higher capacity with a minor effect on the stiffness.

**Clinical significance:**

This paper provides insights into the mechanical behaviour of orthodontic mini-implants, offering guidance to orthodontic practitioners and researchers. Understanding how torque affects stability and performance under different loads informs the selection and design of mini-implants, potentially improving orthodontic treatments and patient outcomes.

## 1 Introduction

Anchorage is defined as resistance to unwanted tooth movement [1]. In orthodontic treatment, anchorage planning is essential to achieving pre-established therapeutic results [2]. Many dental or extraoral appliances have been used [3]. Nevertheless, a small force can be generated, and it could cause undesirable dental movements. It is, therefore, essential to eliminate it through absolute anchorage [4]. Absolute anchorage neutralises the force of equal intensity acting in the opposite direction to the desired tooth movement. This takes the form of balancing all unwanted movements of the anchor unit. This is only possible using anchored teeth or an orthodontic mini-implant (OMI). These two elements use bone in common, which neutralizes unwanted forces [5]. In the 80s of the last century, Creekmore and Eklund proposed using mini-screws to use the bone as an anchor to obtain a maximum anchorage without creating unwanted tooth movements [6]. Since then, OMIs have been increasingly used to obtain an absolute anchorage, as they avoid all the drawbacks of a dental or extraoral anchorage [7]. There is no consensus among the authors either in the design protocol or in the insertion of the OMIs. The failure rate in the literature is approximately 13.5%: a relatively low percentage, which allows us to conclude that OMIs can be used in orthodontic clinical practice, as they are reliable [8]. The success rate of temporary anchorage devices (TADs) mainly depends on aspects enclosed in 3 macro-categories: characteristics dependent on the patient, technical specifications of the OMIs or factors related to the insertion technique [9]. The probability of success of the OMIs is mainly linked to obtaining primary stability [10]. This is understood as the absence of micro-movements of the OMIs following their insertion into the surrounding bone due to a maximum interdigitation between the bone tissue and the OMIs threads. It is primarily influenced by OMI design characteristics, quality and quantity of cortical bone and surgical skills of the operator [11], [12]. The OMI design characteristics include length, diameter, thread depth, width, helix angle and pitch values, thread depth-to-outer diameter ratio, presence of flutes (that allow the screw to penetrate the bone more quickly as they remove the bone fragments that are created when the screw is inserted) and body shape, that can be conical or cylindrical. Length and diameter are essential for obtaining the OMI primary stability [13], [14].

This research contributes novel insights into OMI by comprehensively analyzing their mechanical response under different loading conditions. One of this study's critical novel aspects is using a dedicated testing apparatus with a universal testing machine to evaluate mini-implants installed in pig palates. Using an animal model provides a more clinically relevant and realistic representation of the mechanical behaviour of mini-implants in a biological setting, enhancing the applicability of the findings to orthodontic practice. Moreover, this study investigates seven different types of mini-screws from two manufacturers, providing a diverse range of geometrical characteristics to assess their mechanical performance. Considering various torque values during the testing process adds another element of novelty, revealing the direct impact of torque on the resistance to failure and stiffness of the screws under different loading directions. The comprehensive analysis of the data obtained from pull-out, push-in, and shear tests allows for a thorough evaluation of the screws' performance, enabling orthodontic practitioners and researchers to make informed decisions in selecting the most suitable mini-implants for specific clinical scenarios. Furthermore, the estimation of ultimate shear stress and embedment stiffness values from the conducted tests facilitates the development of simplified finite element models, offering a valuable tool for further understanding the mechanical behaviour of mini-implants and their interactions with bone tissues. Overall, the novelty of this research lies in its multidimensional approach, combining experimental investigations, diverse screw types, torque variations, and finite element modelling to advance the understanding of mini-implant mechanical performance and its implications in orthodontic treatment planning.

## 2 Test description

The authors' ethical consideration to avoid animal sacrifice motivated the selection of pigs as experimental subjects. Therefore, the study utilized pig specimens obtained from a local butcher shop. Seven self-drilling orthodontic mini-screws made of titanium alloy were subjected to testing. The detailed geometrical characteristics of these screws are provided in Table 1. The study considered two manufacturers: BENEfit and MSE. Specifically, the BENEfit system comprised three mini-screws, while the MSE system included four. The threaded diameter of the screws ranged from 1.5 to 2 mm, and the total length varied from 7 to 15.1 mm. The BENEfit screws are made of titanium, while the MSE screws are composed of the Titanium 6Aluminum 4Vanadium alloy. The mechanical characteristics were obtained from tensile tests conducted on each type of screw. The yielding bending moment ($M_{y}$) has been calculated as in Table 1

**Table 1:** List of the tested screws, where *d* is the diameter, *l* the length, *E* is the Young's modulus, *EI* the elastic bending stiffness, *f*_*y*_ the yielding strength and *M*_*y*_ the yielding bending moment. Ti-6Al-4V is the Titanium 6Aluminum 4Vanadium alloy.

Screw type	*d* [mm]	*l* [mm]	Label	Material	*E* [GPa]	EI [N ⋅ mm^2^]	*f*_*y*_ [MPa]	*M*_*y*_ [N ⋅ mm]
BENEfit4plusV	2	7	BENEfit4plusV_2_7	Titanium	105	82425	380	239
BENEfit4plusV	2	9	BENEfit4plusV_2_9	Titanium	105	82425	380	239
BENEfit4plusV	2	11	BENEfit4plusV_2_11	Titanium	105	82425	380	239
MSE_OAS_T1511	1.5	13.1	MSE_OAS_T1511_1.5_13.1	Ti-6Al-4V	108	26825	810	215
MSE_OAS_T1513	1.5	15.1	MSE_OAS_T1511_1.5_15.1	Ti-6Al-4V	108	26825	810	215
MSE_OAS_T1811	1.8	13.1	MSE_OAS_T1511_1.8_13.3	Ti-6Al-4V	108	55624	810	371
MSE_OAS_T1813	1.8	15.1	MSE_OAS_T1511_1.8_15.1	Ti-6Al-4V	108	55624	810	371

**Figure 1:**
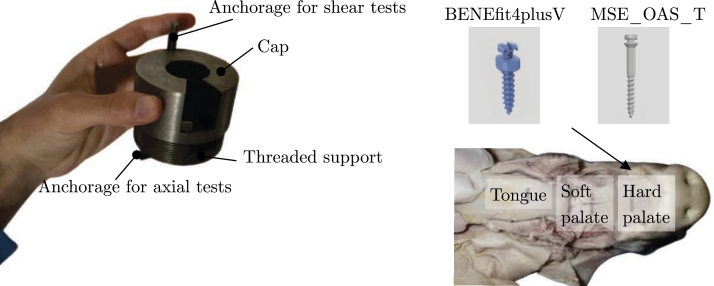
View of the steel artefact used for mechanical tests.

The mini-screws were inserted into the pigs' hard palate. To facilitate their placement, the authors prepared pieces of the hard palate that could be fitted into the steel template shown in Fig. 1. This steel artefact was explicitly designed for conducting mechanical tests on the mini-screws.

The steel template consists of two components: a steel cap and a threaded support. The section of the hard palate is inserted into the threaded support and securely held in place by the steel cap, which is equipped with 20 mm flanges. The cap features a circular opening for installing the mini-screws.

Moreover, the steel device includes two anchorages that enable its connection to the jaws of the testing machine. The mini-screws were positioned using three insertion torques: 25 Ncm, 30 Ncm, and 35 Ncm. The insertion site was carefully selected within the available space inside the support, and the screws were inserted perpendicularly to the perpendicular plate of the maxillary bone. Before each measurement, the torque gauge was appropriately calibrated.

For screw insertion, the authors utilized the NSK handpiece iSD900. The tests were conducted using the ME-8236 materials testing apparatus manufactured by PASCO. This device enables the measurement of force and displacement in various materials subjected to stretching, compression, shearing, or bending.

The PASCO Materials Testing Machine incorporates a built-in load cell (strain gauge transducer) capable of measuring forces up to 7100 N. Additionally, an optical encoder module accurately measures the displacement of the load bar. The load bar can be adjusted using a crank gear connected to two translation screws.

To record, display, and analyze the data, the authors utilized the PASCO PASSPORT Compatible Interface with the PASCO Data Collection Software. The sensor cable from the testing machine was connected to the PASPORT input port, while the PASCO Interface was connected to a laptop's USB port via a USB link.

For each test, nominal stress and stretch values were computed. The loading protocol implemented was displacement-controlled, utilizing a loading rate of 0.1 mm/s.

**Figure 2:**
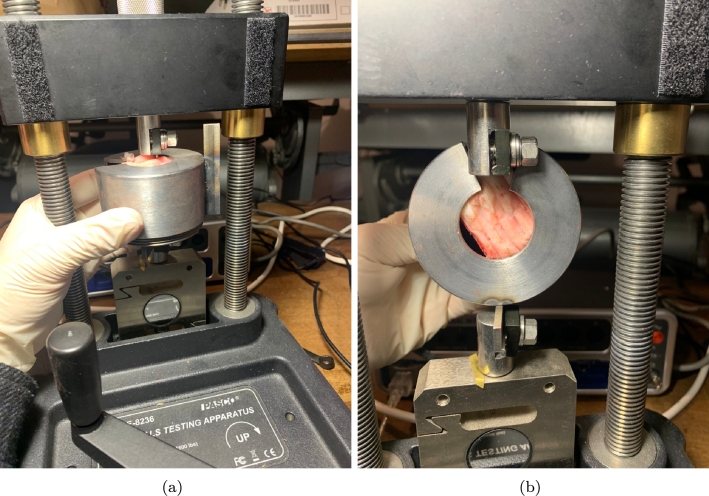
View of the axial (a) and shear tests (b).

Furthermore, Fig. 2 visually represents the axial and shear test apparatus.

By adjusting the orientation of the steel artefact, it becomes feasible to evaluate the mechanical performance of the screws in two orthogonal directions. To estimate the capacity of the screws, the authors estimated the load-displacement curves. The capacity is determined by identifying the maximum force achieved during the test. Additionally, the secant stiffness (Eq. (1)) was defined and calculated as follows:

(1) $$k_{sec}=\frac{F_{max}}{d_{max}}$$

where $k_{sec}$ is the secant stiffness, $F_{max}$ is the maximum force and $d_{max}$ is the displacement corresponding to the peak force value.

The authors carried out 189 tests resulting from the following cross-analysis shown in Eq. (2):

(2) n=7Screw Types×3Loading Directions×3Insertion Torques×3Test Repetitions=189

## 3 Results

The results section is structured into three distinct subsections, each focusing on a specific type of test: pull-out, push-in, and shear tests, respectively.

### 3.1 Pull-out tests

**Figure 3:**
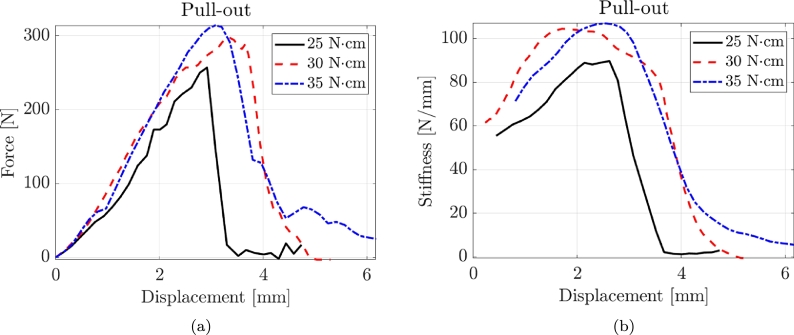
Typical force-displacement and stiffness-displacement curves for pull-out tests with three torque values.

Fig. 3 shows three typical force-displacement and stiffness-displacement curves for pull-out tests corresponding to the three torque values. Pull-out tests exhibit a prevalent linear behaviour until failure. Accordingly, the value of the secant stiffness is also pretty constant from the early to final stages of deformation.

Table 2 presents the experimental results of the pull-out tests. The results are organized into three subsections, representing different levels of insertion torque: T = 25 Ncm, T = 30 Ncm, and T = 35 Ncm. For each subsection, the table provides the following information for each tested sample: the label, representing the identification of the screw; the maximum force ($F_{max}$) measured in N; the coefficient of variation (CoV), which indicates the variability of the measurements; and the secant stiffness ($k_{sec}$) in N/mm.

**Table 2:** Experimental results of the pull-out tests where CoV is the Coefficient of Variation.

**Table:** Pull-out T = 25 Ncm Label $F_{max}$ [N] CoV $k_{sec}$ [N/mm] CoV BENEfit4plusV_2_7 303.65 0.09 134.26 0.09 BENEfit4plusV_2_9 383.08 0.07 199.56 0.10 BENEfit4plusV_2_11 576.19 0.09 203.25 0.10 MSE_OAS_T1511_1.5_13.1 446.94 0.05 144.41 0.09 MSE_OAS_T1511_1.5_15.1 609.12 0.06 134.10 0.12 MSE_OAS_T1511_1.8_13.3 649.54 0.12 169.50 0.05 MSE_OAS_T1511_1.8_15.1 632.40 0.14 197.46 0.08
**Table:** Pull-out T = 30 Ncm Label $F_{max}$ [N] CoV $k_{sec}$ [N/mm] CoV BENEfit4plusV_2_7 423.64 0.14 96.78 0.07 BENEfit4plusV_2_9 533.80 0.12 189.56 0.08 BENEfit4plusV_2_11 597.90 0.08 172.52 0.08 MSE_OAS_T1511_1.5_13.1 546.41 0.14 193.23 0.15 MSE_OAS_T1511_1.5_15.1 648.51 0.14 232.24 0.15 MSE_OAS_T1511_1.8_13.3 631.39 0.09 275.29 0.06 MSE_OAS_T1511_1.8_15.1 683.27 0.12 245.94 0.10
**Table:** Pull-out T = 35 Ncm Label $F_{max}$ [N] CoV $k_{sec}$ [N/mm] CoV BENEfit4plusV_2_7 390.35 0.09 130.17 0.08 BENEfit4plusV_2_9 546.19 0.10 172.44 0.05 BENEfit4plusV_2_11 684.58 0.12 164.40 0.11 MSE_OAS_T1511_1.5_13.1 629.22 0.12 235.55 0.14 MSE_OAS_T1511_1.5_15.1 741.01 0.08 200.67 0.12 MSE_OAS_T1511_1.8_13.3 791.19 0.11 290.55 0.14 MSE_OAS_T1511_1.8_15.1 736.38 0.10 248.78 0.11

For the T = 25 Ncm pull-out tests, the maximum force ($F_{max}$) ranges from 303.65 N to 649.54 N, with varying coefficients of variation (CoV) between 0.05 and 0.14. The secant stiffness ($k_{sec}$) spans from 134.10 N/mm to 203.25 N/mm, with CoV values ranging from 0.09 to 0.12. In the T = 30 Ncm pull-out tests, the $F_{max}$ values range from 423.64 N to 683.27 N, with CoV ranging from 0.08 to 0.14. The $k_{sec}$ values range from 96.78 N/mm to 275.29 N/mm, with a CoV of 0.09. For the T = 35 Ncm pull-out tests, the $F_{max}$ values range from 390.35 N to 791.19 N, with CoV varying from 0.09 to 0.12. The $k_{sec}$ values range from 130.17 N/mm to 290.55 N/mm, with a CoV of 0.10.

The torque applied during the testing process has a direct impact on the performance of the screws. As the torque increases, the maximum force measured during the pull-out tests tends to increase. This indicates that higher torque values result in higher resistance to extraction for the screws. The torque also influences the secant stiffness of the screws. Increasing the torque generally leads to higher stiffness values. This suggests that higher torque produces stiffer screws, which exhibit less deformation or displacement under applied loads. The effect of torque on CoV may vary depending on the specific test conditions and sample characteristics. In some cases, higher torque values may lead to increased variability in the results, while in other cases, the variability may decrease or remain relatively consistent.

Regarding the geometrical properties of the screws, increasing the diameter of the screws generally results in higher maximum forces and stiffness values during pull-out tests. This is because a larger diameter provides a larger contact area and improved load distribution, leading to increased resistance to extraction. A larger diameter also tends to decrease the risk of screw failure or damage, as it can better withstand applied loads and reduce the stress concentration at the insertion site. The length of the screws affects their mechanical performance in terms of maximum force and stiffness. Longer screws tend to provide increased resistance to extraction and higher maximum forces. The longer length allows for a deeper and more secure anchorage within the bone or substrate. However, it is essential to consider the anatomical constraints and available space when choosing the screw length. Inserting excessively long screws can lead to complications, such as damage to adjacent structures or interference with the desired treatment outcome. It is worth noting that the specific effects of diameter and length can also depend on other factors, such as the material properties of the screws, the bone quality or substrate characteristics, and the applied load conditions. Therefore, careful consideration should be given to selecting the appropriate diameter and length of the screws based on the specific clinical or experimental requirements to ensure optimal performance and stability.

### 3.2 Push-in tests

**Figure 4:**
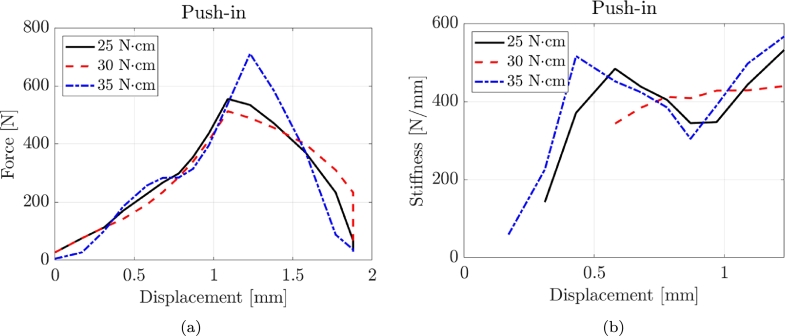
Typical force-displacement and stiffness-displacement curves for compression tests with three torque values.

Fig. 3 illustrates three representative force-displacement and stiffness-displacement curves obtained from the compression tests, corresponding to the three torque values. It is observed that the pull-out tests demonstrate a more ductile behaviour, characterized by an increasing stiffness as the deformation values become higher. Table 3 and Fig. 4 present the experimental results of the compression tests, providing information on the performance of the tested screws. The failure mode did not involve the buckling of the screws with a typical push-in failure. In the compression T = 25 Ncm tests, the maximum force ($F_{max}$) ranges from 497.89 N to 961.77 N, with coefficients of variation (CoV) between 0.05 and 0.15. The secant stiffness ($k_{sec}$) values span from 792.10 N/mm to 1517.04 N/mm, with CoV values ranging from 0.06 to 0.13. For the Compression T = 30 Ncm tests, the $F_{max}$ values range from 346.43 N to 652.68 N, with CoV varying from 0.06 to 0.15. The $k_{sec}$ values range from 631.22 N/mm to 1226.49 N/mm, with CoV values ranging from 0.05 to 0.14. In the Compression T = 30 Ncm tests, the $F_{max}$ values range from 589.51 N to 1229.14 N, with CoV varying from 0.05 to 0.15. The $k_{sec}$ values range from 896.25 N/mm to 1740.99 N/mm, with CoV values ranging from 0.06 to 0.12. The maximum force and stiffness values obtained from the compression tests are significantly higher than the pull-out tests.

**Table 3:** Experimental results of the compression tests where CoV is the Coefficient of Variation.

**Table:** Compression T = 25 Ncm Label $F_{max}$ [N] CoV $k_{sec}$ [N/mm] CoV BENEfit4plusV_2_7 497.89 0.08 792.10 0.13 BENEfit4plusV_2_9 662.00 0.10 948.39 0.08 BENEfit4plusV_2_11 864.66 0.07 1211.86 0.06 MSE_OAS_T1511_1.5_13.1 739.45 0.15 1135.80 0.08 MSE_OAS_T1511_1.5_15.1 814.84 0.15 1178.47 0.10 MSE_OAS_T1511_1.8_13.3 932.37 0.06 1269.02 0.13 MSE_OAS_T1511_1.8_15.1 961.77 0.05 1517.04 0.07
**Table:** Compression T = 30 Ncm Label $F_{max}$ [N] CoV $k_{sec}$ [N/mm] CoV BENEfit4plusV_2_7 346.43 0.06 631.22 0.05 BENEfit4plusV_2_9 410.87 0.10 818.16 0.11 BENEfit4plusV_2_11 561.11 0.06 941.16 0.10 MSE_OAS_T1511_1.5_13.1 466.86 0.15 811.98 0.14 MSE_OAS_T1511_1.5_15.1 531.28 0.14 995.18 0.15 MSE_OAS_T1511_1.8_13.3 556.26 0.06 994.76 0.13 MSE_OAS_T1511_1.8_15.1 652.68 0.06 1226.49 0.14
**Table:** Compression T = 30 Ncm Label $F_{max}$ [N] CoV $k_{sec}$ [N/mm] CoV BENEfit4plusV_2_7 589.51 0.08 896.25 0.08 BENEfit4plusV_2_9 803.87 0.15 1062.55 0.06 BENEfit4plusV_2_11 944.96 0.08 1376.00 0.06 MSE_OAS_T1511_1.5_13.1 864.87 0.06 1225.31 0.10 MSE_OAS_T1511_1.5_15.1 905.56 0.05 1440.93 0.12 MSE_OAS_T1511_1.8_13.3 925.85 0.06 1427.92 0.07 MSE_OAS_T1511_1.8_15.1 1229.14 0.08 1740.99 0.09

### 3.3 Shear tests

**Figure 5:**
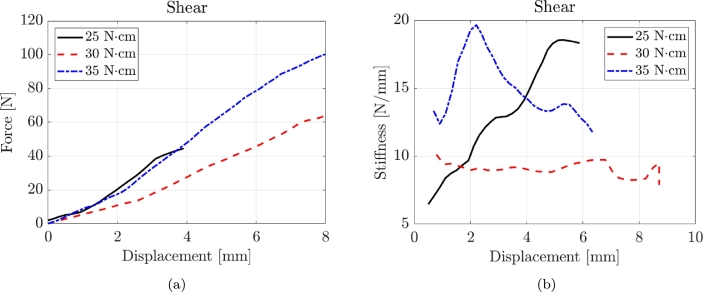
Typical force-displacement and stiffness-displacement curves for shear tests with three torque values.

Fig. 5 displays three representative force-displacement and stiffness-displacement curves obtained from shear tests, each corresponding to different torque values. The shear tests demonstrate a predominant linear behaviour throughout the testing process until failure occurs. Consequently, the secant stiffness maintains a relatively constant value from the initial stages of deformation to the final stages.

Table 4 presents the experimental results of the shear tests.

**Table 4:** Experimental results of the shear tests where CoV is the Coefficient of Variation.

**Table:** Shear T = 25 Ncm Label $F_{max}$ [N] CoV $k_{sec}$ [N/mm] CoV BENEfit4plusV_2_7 13.09 0.13 18.75 0.07 BENEfit4plusV_2_9 23.89 0.14 23.18 0.11 BENEfit4plusV_2_11 30.97 0.10 26.37 0.10 MSE_OAS_T1511_1.5_13.1 28.78 0.12 27.26 0.10 MSE_OAS_T1511_1.5_15.1 25.79 0.06 25.88 0.12 MSE_OAS_T1511_1.8_13.3 25.86 0.12 32.61 0.14 MSE_OAS_T1511_1.8_15.1 37.66 0.13 36.51 0.13
**Table:** Shear T = 30 Ncm Label $F_{max}$ [N] CoV $k_{sec}$ [N/mm] CoV BENEfit4plusV_2_7 15.53 0.08 17.27 0.12 BENEfit4plusV_2_9 19.72 0.13 24.40 0.11 BENEfit4plusV_2_11 30.92 0.08 28.45 0.13 MSE_OAS_T1511_1.5_13.1 23.84 0.08 26.35 0.12 MSE_OAS_T1511_1.5_15.1 33.53 0.08 32.24 0.10 MSE_OAS_T1511_1.8_13.3 30.68 0.14 32.27 0.14 MSE_OAS_T1511_1.8_15.1 42.08 0.11 36.61 0.10
**Table:** Shear T = 35 Ncm Label $F_{max}$ [N] CoV $k_{sec}$ [N/mm] CoV BENEfit4plusV_2_7 22.92 0.06 18.60 0.07 BENEfit4plusV_2_9 28.30 0.10 26.52 0.07 BENEfit4plusV_2_11 32.24 0.06 30.21 0.08 MSE_OAS_T1511_1.5_13.1 27.05 0.11 29.87 0.11 MSE_OAS_T1511_1.5_15.1 35.08 0.05 29.06 0.07 MSE_OAS_T1511_1.8_13.3 32.37 0.13 30.87 0.12 MSE_OAS_T1511_1.8_15.1 37.65 0.09 41.09 0.05

For the shear T = 25 Ncm tests, the maximum force ($F_{max}$) values range from 13.09 N to 37.66 N, with coefficients of variation (CoV) between 0.06 and 0.13. The secant stiffness ($k_{sec}$) values span from 18.60 N/mm to 36.51 N/mm, with CoV values ranging from 0.07 to 0.14. In the Shear T = 30 Ncm tests, the $F_{max}$ values range from 15.53 N to 42.08 N, with CoV varying from 0.08 to 0.13. The $k_{sec}$ values range from 17.27 N/mm to 36.61 N/mm, with CoV values ranging from 0.10 to 0.14. For the shear T = 35 Ncm tests, the $F_{max}$ values range from 22.92 N to 37.65 N, with CoV varying from 0.05 to 0.10. The $k_{sec}$ values range from 18.60 N/mm to 41.09 N/mm, with CoV values ranging from 0.05 to 0.12.

As expected, increasing the diameter of the screws generally leads to higher maximum forces and stiffness values across all test types (pull-out, compression, and shear). A larger diameter provides a larger contact area and improved load distribution, increasing resistance to shear forces.

### 3.4 ANOVA tests

The authors conducted six ANOVA tests to assess how diameter, length, and torque influence the maximum force and the secant stiffness under the three loading conditions (pull-out, push-in, and shear tests). The ANOVA tests involve comparing the variance in the mechanical properties under different conditions to determine if the variation can be attributed to the tested variables or if it's just due to random variation [15]. Table 5 lists the results of the ANOVA tests.

**Table 5:** ANOVA Test Results for *F*_*max*_ and *k*_*sec*_. “Sum Sq.” is the sum of squares; “d.f.” stands for degrees of freedom; “Mean Sq.” stands for the mean sum of squares; “F” is the F-statistic and is a ratio of the Mean Squares from different sources of variation; “Prob>F” represents the p-value associated with the F-statistic.

Variable	Test	Sum Sq.	d.f.	Mean Sq.	F	Prob>F	Sum Sq.	d.f.	Mean Sq.	F	Prob>F
		$F_{max}$ [N]					$k_{sec}$ [N/mm]				
Diameter	Pull-out	21080.7	1	21080.7	9.08	0.01	6879.4	1	6879.4	5.57	0.0346
Length		102222.1	3	34074	14.68	0.0002	8272.2	3	2757.41	2.23	0.133
Torque		60197	2	30098.5	12.97	0.0008	5653.2	2	2826.6	2.29	0.1408

Diameter	Push-in	72884.8	1	72884.8	20.06	0.0006	160672.6	1	160672.6	42.24	2.01E-05
Length		177410.6	3	59136.9	16.27	0.0001	372768.8	3	124256.3	32.67	2.54E-06
Torque		567436.8	2	283718.4	78.07	0	546922.2	2	273461.1	71.89	9.36E-08

Diameter	Shear	86.56	1	86.564	8.06	0.014	128.707	1	128.707	29.2	0.0001
Length		458.1	3	152.7	14.21	0.0002	199.111	3	66.37	15.06	0.0002
Torque		64.41	2	32.203	3	0.085	17.578	2	8.789	1.99	0.1757

All three variables show statistical significance regarding the capacity under pull-out, with diameter showing a minor influence (p = 0.01) compared to length and torque. Conversely, when considering $k_{sec}$, only the diameter has a significant impact (p = 0.0346), though less pronounced than its effect on $F_{max}$. Length and torque do not significantly affect the secant stiffness during pull-out tests (p > 0.1). For the push-in tests, diameter, length, and torque all significantly affect the maximum force, with the torque showing an exceptionally strong association (p-value approaching 0). Diameter's influence on $F_{max}$ remains significant in shear tests (p = 0.014), though it is more pronounced in the stiffness with a p-value of 0.0001. Length retains its significant influence on $F_{max}$ (p = 0.0002), but like torque, it does not significantly impact $k_{sec}$ (p > 0.1). In conclusion, the ANOVA results show that torque is particularly influential in push-in tests for both dependent variables. At the same time, the length appears to be more critical in pull-out tests for $F_{max}$. Diameter's influence is consistent across all tests but is especially significant for $k_{sec}$ in shear tests.

## 4 Discussion

To ensure the applicability of the findings, the authors extrapolated the ultimate shear stress and subgrade stiffness values from all conducted tests. As detailed in the subsequent section, the obtained data can be used for developing simplified finite element (FE) models of the installations. In particular, the expressions for the ultimate shear stress (Eq. (3)) and embedment stiffness (Eq. (4)) for push-in and pull-out are:

(3) $$\tau_{f}=\frac{2F_{max}}{\pi dl_{1}}\;\;\text{[MPa]}$$

(4) $$c=\frac{2k_{sec}}{\pi dl_{1}}\;\;\text{[N/mm}{}^{3}\text{]}$$

where *d* is the screw diameter and $l_{1}$ the threaded length.

Table 6 presents the average values of the ultimate shear stress and embedment stiffness obtained from the tested screws, categorized by different torque values and loading directions.

**Table 6:** Average values of the ultimate shear stress and embedment stiffness for the tested screws for different torque values and loading directions.

Torque [N ⋅ cm]	Pull-out		Push-in		Shear	
	$\tau_{f}$ [MPa]	*c* [N/mm^3^]	$\tau_{f}$ [MPa]	*c* [N/mm^3^]	$\tau_{f}$ [MPa]	*c* [N/mm^3^]
25	7.79	2.67	12.06	17.47	1.64	0.42
30	9.04	2.71	10.76	14.23	3.43	0.42
35	9.52	3.09	13.56	19.65	3.82	0.45

Table 6 provides the average values of the ultimate shear stress ($\tau_{f}$) and embedment stiffness (*c*) for the tested screws, considering different torque values and loading directions. Observing the pull-out loading direction, it can be observed that as the torque increases from 25 N ⋅ cm to 35 N ⋅ cm, the ultimate shear stress ($\tau_{f}$) increases, indicating higher resistance to failure. The embedment stiffness (*c*) also exhibits an increasing trend, suggesting a stiffer response of the screw when subjected to pull-out forces. The ultimate shear stress ($\tau_{f}$) and the embedment stiffness (*c*) show fluctuations with different torque values in the compression loading direction. With increasing torque, the ultimate shear stress ($\tau_{f}$) is almost constant regarding the shear direction. The embedment stiffness (*c*) remains relatively constant across different torque values, indicating a consistent level of stiffness when subjected to shear forces.

**Figure 6:**
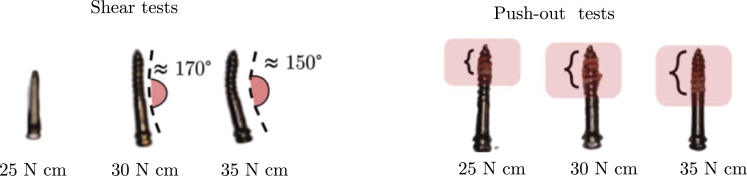
Typical failure mechanisms observed in the screws. The pictures refer to BENEfit4plusV_2_11.

Fig. 6 manifests the beneficial effects of increasing torque. In the case of shear tests, a higher torque leads to a higher embedded, possibly causing the modification of the failure mode (screw or embedment failure). The highest torque is associated with a higher screw deformation until failure.

**Figure 7:**
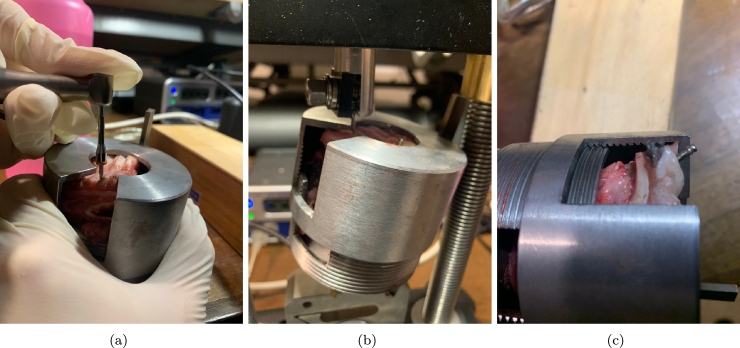
(a) Screw insertion and typical failure observed in the specimens under (b) pull-out and (c) shear forces. The pictures refer to BENEfit4plusV_2_11.

Fig. 7 displays the specimens after failure due to (b) pull-out and (c) shear forces, where the pictures refer to BENEfit4plusV_2_11.

The same occurs for the axial response. A higher torque determines a higher embedment manifested by the bone tissue remaining in the screw threads. Higher torque is associated with more threads filled with bone tissues.

**Figure 8:**
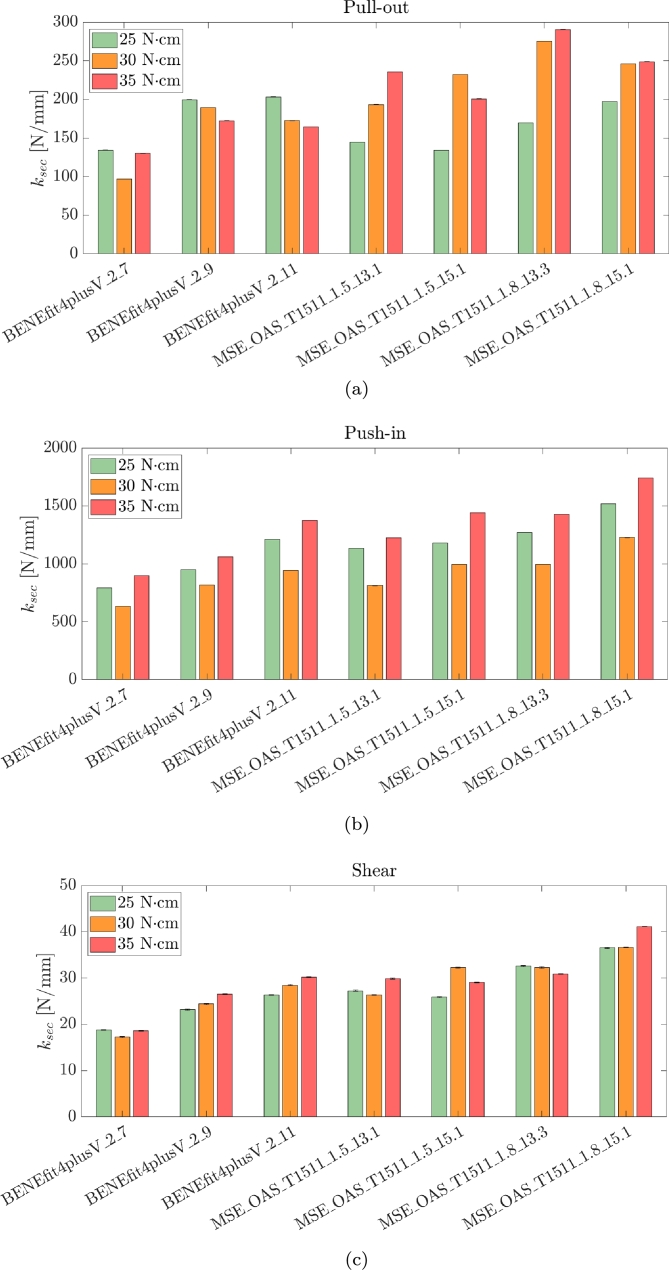
Bar plot of the secant stiffness valued for all tested configurations and the three loading scenarios: (a) pull-out, (b) push-in and (c) shear tests.

Fig. 8 resumes the results of all tested specimens in terms of secant stiffness with bar plots.

The results obtained in this study align with and complement the existing literature on the mechanical behaviour of mini-implants. Several studies have investigated the pull-out, push-in, and shear characteristics of mini-implants under different loading conditions. Compared with previous research, our study's findings confirm the significant influence of torque on the mechanical performance of mini-implants. The observed increase in ultimate shear stress and embedment stiffness with higher torque values aligns with previous studies that have highlighted the importance of adequate torque insertion to achieve better stability and resistance to failure

Brinley et al. conducted a comparative study of OMI with different pitches: 1.00 mm, 1.25 mm, and 0.75 mm. They also compared OMIs with three longitudinal flutes to those without flutes. The study involved testing for maximum placement torque and pull-out strength in both synthetic and cadaver bone. Their findings indicated that decreased MSI pitch leads to increased pull-out strength. Furthermore, the presence of fluting was observed to enhance both placement torque and pull-out strength [16]. Currently, available OMIs exhibit a variety of diameters, lengths, body designs, and thread shapes. They can be fabricated from different alloys. Additionally, the tip design of these OMIs can vary: they are considered self-drilling if equipped with a cutting tip, whereas they are termed self-tapping when featuring a non-cutting tip, necessitating the creation of a pilot hole at the surgical site.

The study by Sabley compared steel screws with titanium screws, examining their insertion at various angles in the upper jaw (30°, 45°, 60°, 75°, and 90°). The authors found no significant differences between the two types of Orthodontic Mini-Implants (OMIs) across these angles, suggesting that both types are equally viable for orthodontic clinical practice [17]. Heo et al. investigated the insertion of tapered OMIs into thick cortical bone at angled predrillings. They concluded that these OMIs achieve better primary stability due to higher maximum insertion torque and comparable total insertion energy values [18]. The research conducted by Suzuki et al. revealed that self-drilling mini-screws offer significantly greater primary stability compared to predrilled OMIs. Moreover, these self-drilling screws exhibited considerably less osseointegration, facilitating safer removal and reducing the likelihood of fracture [19]. Conical-shaped mini-implants have been shown to provide more stability due to closer contact with bone tissue [20], [21]. However, Siegele and Soltesz noted that these implants generate higher crestal stresses than cylindrical ones of the same size [22].

Barros et al. employed Scanning Electron Microscopy (SEM) to investigate the mechanical strength differences between stainless steel mini-implants (SS-MI) and titanium alloy mini-implants (TA-MI) with diameters ranging from 1.2 mm to 1.8 mm. These implants were inserted into high-density artificial bone blocks. Their findings revealed that the stainless steel OMIs exhibited enhanced strength, being 13.2% and 20.2% more resistant to torsional force and deflection, respectively. This suggests that the risk of fracture can be mitigated without increasing the screw diameter [23]. Various quantitative methods are available to assess stability, including Periotest, resonance frequency analysis, pullout tests, and recording of insertion/removal torque [24]. Insertion torque is crucial for achieving optimal primary stability [24], [25]. The consensus in the literature indicates a maximum insertion torque (MIT) value ranging between 5 and 10 N ⋅ cm [24], [25], [26], [27]. Torques exceeding these values might cause cortical bone fracture and bone resorption, both of which are factors that can lead to screw failure [26].

Osseointegration is not considered necessary for Orthodontic Mini-Implants (OMIs), as it could complicate their removal, necessitating increased removal torque values by the orthodontist [7], [28]. A high removal torque might result in the fracture of the OMI [28]. It has been observed that the immediate application of force to the OMI after its insertion into the bone can reduce the likelihood of osseointegration [29]. However, it is noted that partial osseointegration may still occur, creating varying degrees of bone-screw union. In fact, recent clinical and experimental studies have shown that implants, even those immediately loaded, can become partially osseointegrated, demonstrating varying levels of contact [30], [31], [32]. The degree of osseointegration in conventional implants can be assessed based on the extent of the removal torque required [33].

## 5 FE model of the BENEfit mini-implant

Finite Element (FE) models enable clinicians to predict and anticipate potential challenges associated with tooth movements before they manifest in the patient's oral cavity. The authors developed a high-fidelity 3D model of the oral cavity to simulate the mechanical response of the BENEfit mini-implant.

Three-dimensional (3D) rendering plays a crucial role in orthodontics by providing orthodontists and dental professionals with advanced tools for diagnosis, treatment planning, and patient communication [34], [35], [36]. This paper used a 3D rendering of the oral cavity, already available through Ansys, for the analysis.

The authors chose the BENEfit mini-implant due to its advantages in dental and orthodontic applications.

• Versatility: The BENEfit mini-implant is known for its versatility and multiple applications in dentistry. It can be used for various purposes, including supporting dental prosthetics, acting as an orthodontic anchorage, or offering temporary stabilisation during dental procedures.
• Size and Design: The BENEfit mini-implant is compact and allows easy insertion and placement in the oral cavity. This feature can be advantageous when considering patient comfort and minimizing tissue trauma during implantation.
• Clinical Experience and Research: The BENEfit mini-implant has significant clinical experience and research supporting its effectiveness and reliability.

The oral cavity was modelled using solid elements to reduce the computational burden. In contrast, the mini-screws were modelled as Winkler beams with subgrade stiffness, as specified in Table 6. The model was developed using ANSYS, a widely used software for engineering simulations. Table 7 lists the elastic material and interface properties of the anatomic model.

**Table 7:** Elastic material and interface properties of the anatomic models after [37].

**Table:** Material Young's modulus E (GPa) Poisson's ratio *ν* Yielding strength (MPa) Trabecular bone 1.5 0.3 2 Cortical bone 14.7 0.3 133 Tooth 20.7 0.3 - PDL 6.89*10-^5 0.45 Miniscrew 114 0.34 880
**Table:** Interface Friction coefficient Miniscrew-bone 0.3 PDL-tooth Bonded PDL-bone Bonded Tooth-bone 0.37

Usually, orthodontic forces are between 0.3 and 4.0 N [38]. In the present case study, the authors used values that far exceed the biologically acceptable range to analyze the general behaviour of the screw under load conditions. It would be clinically impossible to exert a significant force capable of causing immediate displacement of the screw. The forces measured during pullout tests are usually greater than 100 N, aiming to analyze the absolute mechanical properties. The authors aim to simulate a clinically reproducible orthodontic movement. Therefore, they assumed a force equal to 1 N.

**Figure 9:**
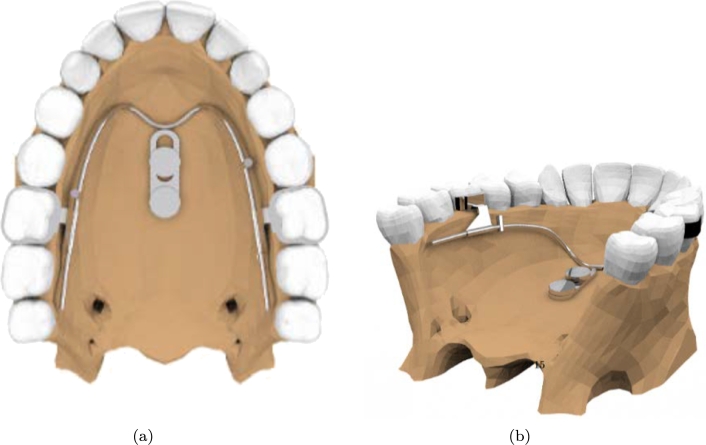
Views of the 3d model imported in ANSYS.

Fig. 9 shows a view of the 3D model imported in ANSYS.

The study found that when a 1 N orthodontic force was applied, it generated an 8 N force on the screw of the BENEfit mini-implant. This observation has important implications for the mechanical behaviour and response of the mini-implant system. The implication is that the force acting on the screw does not reduce but instead increases significantly compared to the applied force. This phenomenon can be attributed to the leverage response of the mini-implant, where the screw acts as the pivot point. In this leverage system, the 1 N force applied at one end generates a much larger force, approximately 8 N, at the screw end. This leverage effect is essential when designing and applying orthodontic forces using mini-implants. A relatively small force applied at a distant location can produce a much greater force at the screw, allowing for effective orthodontic treatment and tooth movement. Understanding this leverage response helps orthodontists and researchers estimate and control the forces exerted on the mini-implant, ensuring optimal treatment outcomes and minimizing the risk of implant failure or complications.

**Figure 10:**
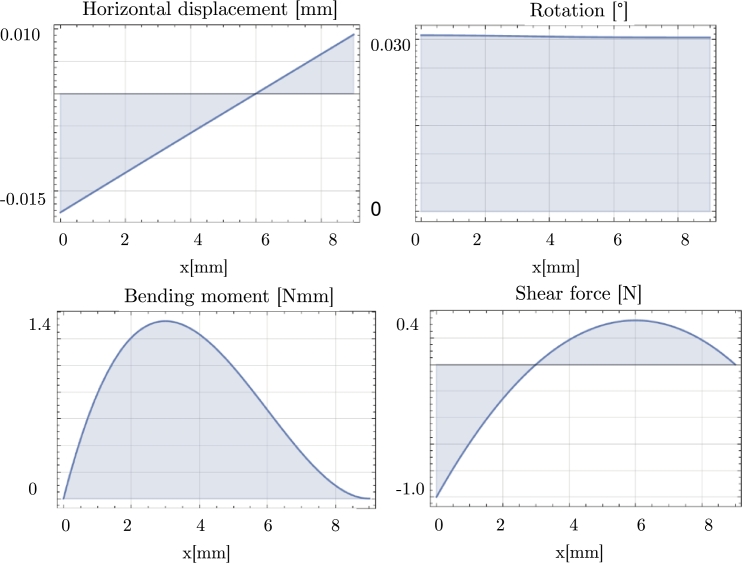
Static response of a mini-screw BENEfit4plusV_2_7 under a 1 N shear force.

To understand the stress distribution along the screw of the BENEfit mini-implant, the authors reported various parameters under a 1 N force applied at the head of the screw: displacement, rotation, bending moment, and shear (see Fig. 10). The results showed that the rotation of the screw was minimal, indicating that it experienced limited rotational movement under the applied force. On the other hand, the displacement was approximately 0.01 mm, suggesting a slight linear movement or deflection of the screw. Table 8 reports the stress values at the screw-bone interface for different types of screws under the 1 N force. By considering these stress values, orthodontists can evaluate the stress level and determine if it falls within acceptable limits to ensure the stability and functionality of the mini-implant during orthodontic treatment.

**Table 8:** Stress values at the bone-miniscrew interface due to 1 N force applied at the screw head.

Average c [N/mm^3^]	Diameter [mm]	Length [mm]	Average stress [N/mm^2^]
0.43	2	7	0.0227
	2	9	0.0177
	2	11	0.0145
	1.5	13.1	0.0162
	1.5	15.1	0.0141
	1.8	13.1	0.0135
	1.8	15.1	0.0117

## 6 Conclusions

1. The study analyzed the mechanical response of orthodontic mini-implants (OMI) under pull-out, push-in, and shear forces, providing valuable insights into their performance under different loading conditions.

2. The research considered various torque values during testing, revealing the direct impact of torque on the resistance to failure and stiffness of the screws under different loading directions.

3. Pull-out tests exhibited a prevalent linear behaviour until failure, while push-in tests showed a more ductile response with increasing stiffness as deformation values increased.

4. Increasing the torque during testing resulted in higher maximum forces and stiffness values, indicating that higher torque values enhance mini-implants' resistance to extraction, compression, and shear forces.

5. The study extrapolated ultimate shear stress and embedment stiffness values from the conducted tests, providing valuable data for constructing simplified finite element models to understand the mechanical behaviour of mini-implants.

6. The obtained data were used to develop high-fidelity 3D finite element models, allowing further assessment of the OMI mechanical performance.

## CRediT authorship contribution statement

**Cristina Valeri:** Writing – review & editing, Writing – original draft, Visualization, Validation, Supervision, Software, Resources, Project administration, Methodology, Investigation, Funding acquisition, Formal analysis, Data curation, Conceptualization. **Angelo Aloisio:** Writing – review & editing, Writing – original draft, Visualization, Validation, Supervision, Software, Resources, Project administration, Methodology, Investigation, Funding acquisition, Formal analysis, Data curation, Conceptualization. **Vincenzo Quinzi:** Visualization, Supervision, Investigation, Conceptualization. **Gianmarco di Stefano:** Visualization, Validation, Supervision, Software, Methodology, Formal analysis, Data curation. **Giuseppe Marzo:** Supervision, Project administration, Funding acquisition.

## Declaration of Competing Interest

The authors declare the following financial interests/personal relationships which may be considered as potential competing interests:

Angelo Aloisio reports article publishing charges was provided by 10.13039/501100006256University of L'Aquila. Angelo Aloisio reports a relationship with University of L'Aquila that includes: employment. If there are other authors, they declare that they have no known competing financial interests or personal relationships that could have appeared to influence the work reported in this paper.

## Data Availability

All data, models, or codes supporting this study's findings are available from the corresponding author upon request.
